# Induction of Apoptosis, G_0_/G_1_ Phase Arrest and Microtubule Disassembly in K562 Leukemia Cells by Mere15, a Novel Polypeptide from *Meretrix meretrix* Linnaeus

**DOI:** 10.3390/md10112596

**Published:** 2012-11-21

**Authors:** Ming Liu, Xiangzhong Zhao, Jin Zhao, Lin Xiao, Haizhou Liu, Cuicui Wang, Linyou Cheng, Ning Wu, Xiukun Lin

**Affiliations:** 1 Institute of Oceanology, Chinese Academy of Science, 7 Nanhai Rd, Qingdao 266071, China; Email: lmouc@hotmail.com (M.L.); zhaojinwang2136@126.com (J.Z.); xiaolin_qd@163.com (L.X.); liuhz0401@163.com (H.L.); Cuicuiwang1122@163.com (C.W.); clyou1976@yahoo.com.cn (L.C.); wuning2283720@126.com (N.W.); 2 Key Laboratory for Rare Diseases of Shandong Province, Institute of Basic Medicine, Shandong Academy of Medical Science, Jinan 250062, China; Email: fzzg.2002@163.com

**Keywords:** *Meretrix meretrix *Linnaeus, Mere15, apoptosis, anticancer

## Abstract

Mere15 is a novel polypeptide from *Meretrix meretrix* Linnaeus with cytotoxicity in solid cancer cells. In this study, we investigated its activity on human K562 chronic myelogenous leukemia cells. Mere15 inhibited the growth of K562 cells with IC_50_ values of 38.2 μg/mL. Mere15 also caused concentration dependent induction of apoptosis, with overproduction of reactive oxygen species and loss of mitochondrial membrane potential. Moreover, Mere15 arrested cell cycle progression at G_0_/G_1_ phase of K562 cells in a concentration dependent manner. In addition, Mere15 caused the disassembly of the microtubule cytoskeleton in K562 cells and inhibited the polymerization of tubulin in a cell free system via interaction with tubulin. We concluded that Mere15 was cytotoxic to K562 leukemia cells and the cytotoxicity was related to the apoptosis induction, cell cycle arrest and microtubule disassembly. These results implied that Merer15 was a broad spectrum anticancer polypeptide, not only cytotoxic to various solid cancer cells but also to the chronic myelogenous leukemia cells. Mere15 may have therapeutic potential for the treatment of leukemia.

## 1. Introduction

Marine organisms are a rich resource for discovering novel anticancer drugs [1,2]. A lot of peptides from marine source display potent anticancer activity [3]. For example, Didemnin, a cyclic depsipeptides isolated from the Caribbean tunicate *Trididemnun solidum*, is the first marine peptide that entered in clinical trials in US for the treatment of cancer [4]. Kahalalide F, a depsipeptide isolated from the herbivorous marine mollusk *Elysia rufescens*, shows strong antitumor activity both in preclinical and clinical studies [5]. Other marine obtained anticancer peptides such as hemiasterlin, dolastatins, cemadotin, soblidotin and aplidine have also entered in clinical trials [4]. The discovery of Dolastatin 10, originally isolated from the Indian Ocean sea hare, *Dolabella auricularia*, has lead to the development of brentuximab vedotin, which has been recently approved by the US Food and Drug Administration for the treatment of Hodgkin’s lymphoma and systemic anaplastic large cell lymphoma [6]. These researches suggest that it is a promising strategy to search anticancer peptides/proteins from the marine organisms.

The marine mollusk *Meretrix meretrix *Linnaeus has been used by traditional Chinese medicine for the treatment of cancer for a long time. Recent studies have shown that certain peptides and/or proteins derived from *M. meretrix* are contributed to their anticancer activity. For example, a peptide from *M. meretrix* has been reported to strongly inhibit the growth of human gastric gland carcinoma cells [7]. In addition, two *M. meretrix* derived glycoproteins, MGP0405 (MW: 9655 Da) [8] and MGP0501 (MW: 15,878 Da) [9], also possess anticancer activity *in vitro*. In our previous studies, we have isolated a novel 15 kDa polypeptide (Mere15) from *M. meretrix*. Mere15 exhibits significant cytotoxicity to several solid cancer cell lines via apoptotic pathway [10]. Moreover, Mere15 inhibits metastasis of human lung cancer A549 cell line via down-regulating matrix metalloproteinases [11]. However, the mode of action of Mere15 induced apoptotic pathways needs to be further elucidated. On the other hand, our previous research was only confined to the solid cancer cell lines; whether Mere15 could also be cytotoxic to hematological malignant tumors remains unknown.

Unlike solid tumors, hematological malignant tumors could not be cured by surgical treatment or radiation therapy and therefore largely depend on the chemotherapy. There have been considerable advances in the treatment of leukemia over the last decade; however, the major obstacle associated in chemotherapy is the resistance of leukemic cells to various chemotherapeutic agents and drug intolerance [12]. To reverse the resistance and deduce the side effects, new therapeutic agents are still needed. In the present study, we evaluated the cytotoxicity of Mere15 on human K562 chronic myelogenous leukemia cells. Moreover, we investigated the possible mechanisms underlying their cytotoxicity.

## 2. Results and Discussion

### 2.1. Mere15 Inhibits K562 Cell Proliferation

**Figure 1:**
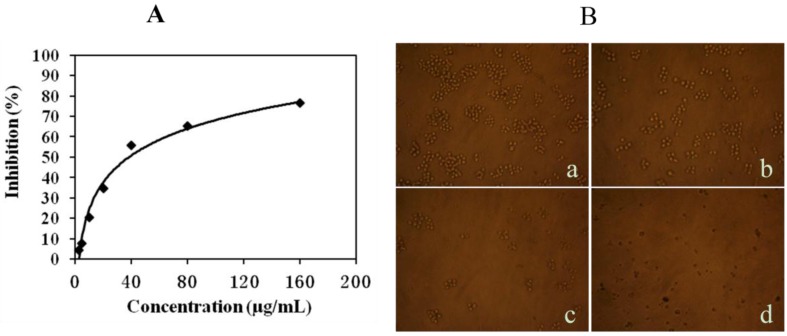
Mere15 inhibits K562 cell proliferation. (**A**) Cytotoxicity of Mere15 against K562 cell lines. Cells in 96 well plates were treated with indicated concentrations of Mere15 for 48 h, and then cell viability was determined by MTT assay as described in Materials and Methods section. (**B**) K562 cells were untreated (a) or treated with 20 (b), 40 (c), and 80 μg/mL (d) Mere15 for 48 h, and observed by inverted microscope.

The cytotoxicity of Mere15 on K562 cell lines was evaluated by MTT assay. As shown in Figure 1A, Mere15 displayed obvious cytotoxicity on K562 cell lines in a concentration dependent manner with the IC_50_ value of 38.2 μg/mL. Treatment with certain concentrations of Mere15 induced decreased population and obvious cell morphological shrinkage (Figure 1B). These results implied that Merer15 was a broad spectrum anticancer polypeptide, not only cytotoxic to various solid cancer cells as reported previously [10], but also to the chronic myelogenous leukemia cells.

### 2.2. Mere15 Induces Apoptosis in K562 Cells

**Figure 2:**
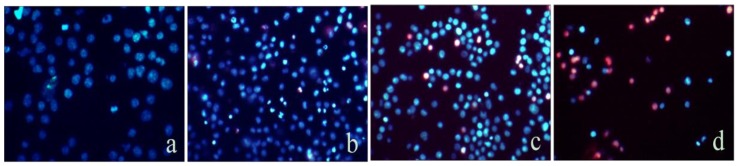
Analysis of apoptosis by double-staining with Hoechst 33342/PI. The K562 cells were untreated (**a**) or treated with 20 (**b**), 40 (**c**), and 80 μg/mL (**d**) of Mere15 for 24 h. Cells were stained with Hoechst33342 and PI, and observed under the fluorescence microscopy.

Since apoptosis accounts for the major mechanism of most anticancer agents, we investigate the apoptosis-inducing activity of Mere15 using Hoechest 33342 and PI double-staining assay. The results showed that the nuclei of untreated K562 cells exhibited diffuse staining of chromatin and no cells were stained by PI nor exhibited brighter blue fluorescence (Figure 2a). With the treatment of Mere15, more nuclei exhibited brighter blue fluorescence, indicating the nuclear condensation in apoptotic cells (Figure 2b–d). Furthermore, with the increasing concentration of Mere15, the number of PI staining cells increased, indicating the increase of the necrotic or advanced apoptotic cells (Figure 2c,d). These results suggested that Mere15 could induce apoptosis in K562 cells in a concentration dependent manner. 

### 2.3. Mere15 Induces ROS Generation

**Figure 3:**
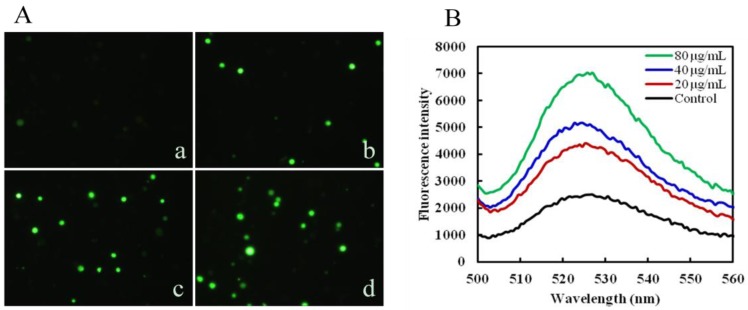
Mere15 induces ROS generation in K562 cells. K562 cells were untreated (a) or treated with 20 (b), 40 (c), and 80 μg/mL (d) Mere15 for 24 h, respectively. Cells were stained with fluorescent probe DCFH-DA (10 μM) for 30 min at 37 °C, and visualized under a fluorescence microscopy (**A**), and detected by fluorescence spectrophotometer, Excitation 488 nm, Emission 525 nm (**B**).

ROS overproduction plays a vital role in apoptosis [13]. To further study whether the Mere15 induced apoptosis has a relationship with the ROS production, the generation of intracellular ROS was analyzed by DCFH-DA. As shown in Figure 3A, an obvious increase of fluorescence intensity was observed after treated with certain concentrations of Mere15 (b, c, d). At the same time, the DCF fluorescence was also measured by fluorescence spectrophotometer to further support the results obtained by fluorescence microscopy. Figure 3B illustrated the emission spectra of cells incubated with DCFH-DA after exposure to Mere15 for 24 h. The emission spectra suggested there’s an increasing generation of ROS after treatment with Mere15. Both these results showed that Mere15 could remarkably enhance the generation of ROS in K562 cells. It is well known that ROS accumulation has a close relationship with caspase signaling pathway [14,15]. In our previous studies, we found that Mere 15 could activate the caspase-3, caspase-9, and PARP, reduce the expression of Bcl-2 and enhance the expression of Bax [10]. The increased accumulation of ROS observed in this study may partly account for the activation of caspase cascade and the subsequent apoptosis. 

### 2.4. Mere15 Disturbs MMP

**Figure 4:**
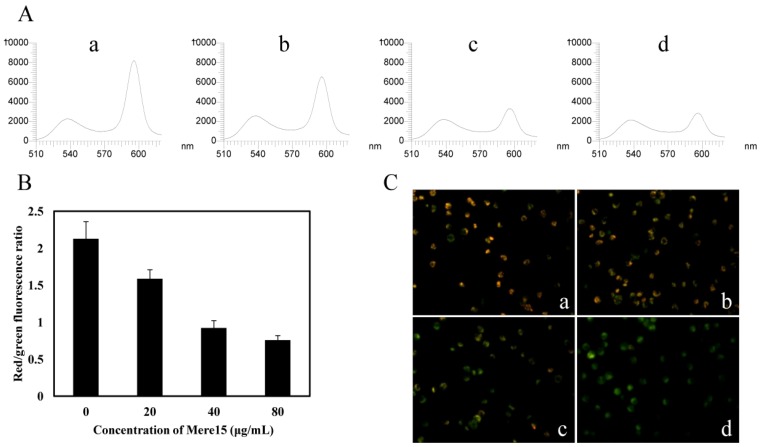
Mere15 induces the loss of MMP in K562 cells. (**A**) JC-1 fluorescence in K562 cells untreated (a) or treated with 20 (b), 40 (c), and 80 μg/mL (d) were analyzed by fluorescence spectrophotometer. The first peak represents the green fluorescence (JC-1 monomer, Excitation 514 nm, Emission 529 nm) intensity and the last peak represents the red fluorescence (JC-1 aggregates, Excitation 585 nm, and Emission 590 nm) intensity. (**B**) Histograms shows ratio of red to green fluorescence intensity of cells treated with various concentrations of Mere15. Values represent the means ± SD. (**C**) Representative merged red-green fluorescent images of JC-1 in K562 cells untreated (a) or treated with 10 (b), 20 (c), and 40 μg/mL (d) Mere15 for 24 h, respectively.

To investigate Mere15 induced dysfunction of mitochondria, the changes of MMP were determined using mitochondrial dye JC-1. As shown in Figure 4A, the fluorescence intensity detected by the fluorescence spectrophotometer showed a decrease in the red fluorescence after treatment with Mere15. The ratio of red to green fluorescence intensity decreased to 0.7 when treated with 80 μg/mL Mere15, as compared with a ratio of 2.1 in the untreated cells (Figure 4B). The decrease in the red fluorescence intensity and increase in the green fluorescence intensity was further confirmed by the observation in fluorescence microscope. As shown in Figure 4C, with the increasing concentration of Mere15, the red fluorescence intensity in K562 cells decreased, while the green fluorescence intensity enhanced gradually. These results revealed Mere15 disturbed MMP and induced mitochondrial dysfunction in K562 cells.

ROS overproduction and ROS-mediated mitochondrial depolarization has been widely reported in other natural products induced apoptosis in cancer cells [16,17,18]. In our previous study, we have found that Mere15 could induce cell death via apoptotic pathway on human lung cancer A549 cells. The present study showed that Mere15 also induced K562 cell death in a similar manner, and ROS overproduction and MMP disruption were all involved in Mere15 induced cell apoptotic pathway. 

### 2.5. Mere15 Induces a G_0_/G_1_ Phase Arrest

**Figure 5:**
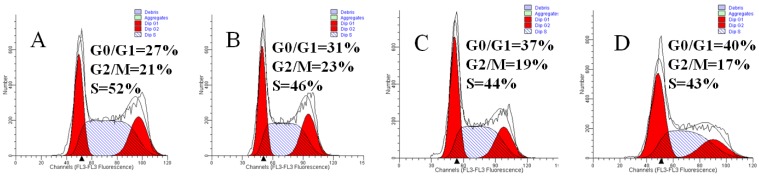
Mere15 induces cell cycle arrest in K562 cells. Cells untreated (**A**) or treated with 20 (**B**), 40 (**C**), and 80 μg/mL Mere15 (**D**) for 24 h and were collected by centrifugation and stained by PI. The DNA contents of the cells were determined with the Aria FACS flow cytometry system, and cell cycle distribution was analyzed with ModFit LT software (Verity Software House. Inc., USA).

To examine whether Mere15 induced effect of anti-proliferation was also associated with cell cycle arrest, cell cycle distribution was analyzed by flow cytometry. As shown in Figure 5, treatment of K562 cells with Mere15 induced accumulation of cells at G_0_/G_1_ phase in a concentration dependent manner. The amount of cells in the G_0_/G_1_ phase increased from 27% in untreated cells to 31%, 37% and 40% in K562 cells treated with 20, 40, and 80 μg/mL Mere15, respectively. These results suggested that Mere15 induced G_0_/G_1_ phase arrest in K562 cells. It should be noted that cells arrested at G_0_/G_1_ phase around Mere15 IC_50_ concentration (80 μg/mL) differs only by 10% from the control. This result indicates that the cell cycle arrest is only one of the effects of Mere15, and possibly other mechanisms are involved in the Mere15 induced proliferation inhibition in K562 cells. Our previous results shows that Mere15 induces G_2_/M phase arrest in A549 cell lines [10], however, this study reveals that the polypeptide induces G_0_/G_1_ arrest in K562 cells. These results suggest that Mere15 blocks the cell cycle in different phase on different cell lines. Similar results were also observed in other anticancer agents induced cell cycle arrest. *p*-methoxyphenyl *p*-toluenesulfonate caused G_2_/M phase arrest in MCF-7 cell lines but G_0_/G_1_ phase arrest in MD-MB-453 cells [19]. *Sepia* ink oligopeptide induced strong G_2_/M phase cell cycle arrest in DU-145 and LNCaP cell lines, however, this oligopeptide induced G_0_/G_1_ phase arrest in PC-3 cells [20]. The mechanisms underlying the differences in different cells remain unclear. However, it is conceivable that the unsimilarity of growth and development between different cells results in the noticeable change on the cell cycle. 

### 2.6. Mere15 Inhibits Tubulin Polymerization both in K562 Cells and in Cell Free System

**Figure 6:**
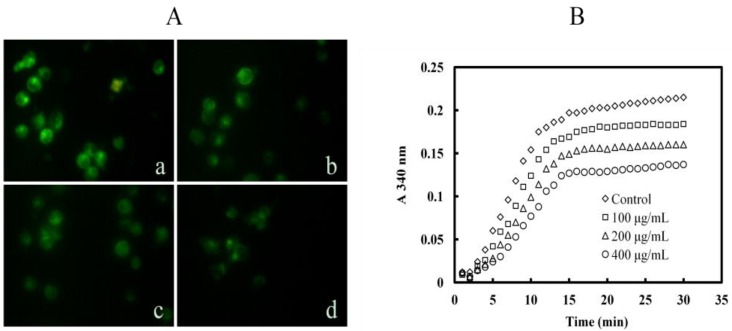
Mere15 inhibits tubulin polymerization. (**A**) Mere15 inhibits the polymerization of cellular α-tubulin. K562 cells were untreated (a) or treated with 20 (b), 40 (c), and 80 μg/mL Mere15 (d) for 24 h. Then cells were collected, fixed, and incubated with mouse monoclonal anti-α-tubulin antibody. Cellular microtubules were observed under fluorescence microscopy. (**B**) Effects of Mere15 on tubulin polymerization. Polymerization of microtubules in various concentrations (0–400 μg/mL) of Mere15 was recorded continuously for 30 min by measuring the absorbance at 340 nm.

To investigate whether the Mere15 induced G_0_/G_1_ phase arrest in K562 cells has a relationship with microtubule polymerization, immunofluorescence of α-tubulin was employed and the result was shown in Figure 6A. The microtubule network in the control cells showed normal organization (a). However, with the treatment of Mere15, the microtubule network was disrupted and diffused in K562 cells (b, c, d). To further investigate whether Mere15 directly inhibits the tubulin polymerization, we next examined the tubulin dynamics in cell free systems. As shown in Figure 6B, Mere15 could remarkably inhibit the tubulin polymerization in a concentration dependent manner. These results suggested that Mere15 could inhibit the tubulin polymerization both in cancer cells and cell free system, which may contribute to the cell cycle arrest and apoptosis activity. Some other anticancer agents like Jaspolide B induce apoptosis in human hepatoma cells. Similar to Mere15, Jaspolide B also affects microtubule-disassembly and arrest cells in G_1_ phase [21]. The underlying mechanism of these agents on microtubule inhibition and G_0_/G_1_ arrest requires further investigations.

### 2.7. Mere15 Interacts with Tubulin *in Vitro*

**Figure 7:**
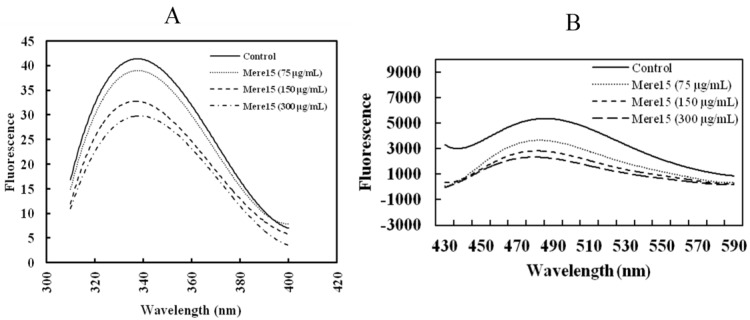
Mere15 interacts with tubulin *in vitro*. (**A**) Mere15 induces intrinsic fluorescence spectra changes of tubulin. Tubulin (2 μM) was incubated with specified concentrations of Mere15 (0–300 μg/mL) for 30 min at 37 °C. The excitation wavelength was 295 nm and emission spectra were acquired by scanning from 300 to 400 nm. (**B**) Changes of tubulin-ANS complex fluorescence by Mere15. Tubulin (2 μM) was incubated for 30 min at 37 °C in the absence or presence of specified concentrations of Mere15 (75, 150, 300 μg/mL). Bis-ANS (15 μM) was then added, and fluorescence was measured after 15 min of incubation at 37 °C (excitation at 400 nm, emission at 430–590 nm) using a Cary Eclipse fluorescence spectrophotometer.

Intrinsic fluorescence quenching was employed to study the potential interaction between Mere15 and tubulin. The fluorescence emission spectrum of tubulin incubated with or without Mere15 was shown in Figure 7A. There are some tryptophan (Trp) residues in Mere15, and the polypeptide also displayed fluorescence emission from 300 to 400 nm. Therefore, the fluorescence interference contributed from different concentrations of Mere15 was deducted, respectively. When tubulin was incubated with Mere15, the fluorescence intensity of tubulin was quenched gradually with the increasing concentrations of Mere15. These results suggest that there is an interaction between Mere15 and tubulin; the interaction results in a microenvironment variation for Trp residues and a conformational change in tubulin. 

To further confirm the interaction between Mere15 and tubulin, we also detected the hydrophobicity of tubulin in both the absence and presence of Mere15. The hydrophobic molecule bis-ANS binds stoichiometrically to tubulin and inhibits microtubule assembly. Bis-ANS is a useful probe in measuring surface hydrophobicity of proteins due to its own hydrophobicity and environmental sensitivity [22]. Mere15 alone gave negligible fluorescence. However, when Mere15 incubated with bis-ANS, a little fluorescence intensities were resulted and this fluorescence was deducted when test the hydrophobicity of tubulin. As shown in Figure 7B, with increasing concentrations of Mere15, the intensity of tubulin-bis-ANS fluorescence was reduced in a concentration dependent manner, suggesting that Mere15 could interact with tubulin and reduce the hydrophobic surface of tubulin.

A large number of substances with anticancer activity interact with tubulin and inhibit microtubule polymerization both *in vitro *and *in vivo*. The *Vinca* alkaloid site and the colchicine site are two important tubulin binding sites and many of the microtubule depolymerizing agents bind to one of these sites [23]. Whether Mere15 could binding to the *Vinca* alkaloid site and the colchicine site, or bind to a novel one on tubulin remains unknown. The exact interaction between Mere15 and tubulin as well as the identification of the binding sites are under way in our lab. Since Mere15 is a polar macromolecule, the underlying mechanisms that Mere15 internalizes into the cells and subsequently disrupts the microtubule network remain to be addressed. Additionally, cells could potentially recover and start dividing again after the remove of the drugs, and more studies are needed to address the sustained period of inhibition of Mere15 on cancer cells. Studies are in progress in our laboratory to confirm whether K562 cells could recover after the remove of Mere15, and if there are synergistic effects between Mere15 and 5-fluorouracil.

## 3. Experimental Section

### 3.1. Materials and Reagents

Mere15 was isolated and purified as described previously [10]. Tubulin was the product of Cytoskeleton (Denver, Co.). Mouse anti-human tubulin monoclonal antibody was purchased from Lab Vision (Saint Louis, MI, USA). Other reagents and kits were the products of Beyond, China. 

### 3.2. Cell Lines and Cell Culture

K562 cells, purchased from ATTCC, were grown on RPMI 1640 (with 300 mg/L glutamine) supplemented with 10% fetal bovine serum and 1× antibiotics at 37 °C in a 5% CO_2_ incubator. 

### 3.3. Cell Viability Assessment

The cytotoxicity of Mere15 was determined by MTT assay [24]. Briefly, cancer cells were untreated or treated with certain concentrations of Mere15 (0–160 μg/mL) for 48 h. MTT (20 μL, 0.5 mg/mL) was added and incubated for another 4 h, and then the supernatant was removed and the dye crystals were dissolved in 200 μL DMSO. Absorbance was measured at 490 nm using a microplate reader (BioTek, USA). The cytotoxicity of Mere15 against K562 cells was expressed as an IC_50_, which was determined from the concentration-response curve.

### 3.4. Hoechst33342/Propidium Iodide (PI) Dual Staining Assays

The apoptotic cells were stained using Hoechst33342/PI double staining as we described previously [25]. The K562 cells were seeded in 6-well plates (2 × 10^5^ cells/well) and treated with certain concentrations of Mere15 (0–80 µg/mL). After incubation for 24 h, the cells were collected and washed with PBS. Cells were stained with Hoechst33342 and PI using the dual staining kit (Beyond, China). Then the cells were spread on slides and observed under the fluorescence microscopy (Zeiss, Germany).

### 3.5. Measurement of Reactive Oxygen Species (ROS) Generation

Generation of ROS was measured by the oxidation-sensitive fluorescent probe DCFH-DA (Beyond, China) according to the manufacturer’s instructions [26]. Briefly, K562 cells were seeded in 6-well plates and treated with certain concentrations of Mere15 (0–80 μg/mL) for 24 h. Then cells were incubated in serum-free culture medium with DCFH-DA (10 μM) for 30 min at 37 °C. Cells were washed with serum-free medium three times and analyzed by fluorescence microscope (Zeiss, Germany) and fluorescence spectrophotometer (F-4500, HITACHI). 

### 3.6. Assessment of Mitochondrial Membrane Potential (MMP)

Mitochondrial membrane potential was determined using Mitochondrial Membrane Potential Detection Kit according to the manufacturer’s instructions (Beyond, China) as described previously [27]. Briefly, K562 cells were seeded in 6-well plates and treated with certain concentrations of Mere15 (0–80 μg/mL) for 24 h. Then the cells were stained with JC-1 for 20 min at 37 °C. MMP were analyzed by fluorescence microscope (Zeiss, Germany) and fluorescence spectrophotometer (F-4500, HITACHI), respectively. The ratio of red to green fluorescence intensity was measured according to the integration of the peaks in the fluorescence spectra. 

### 3.7. Cell Cycle Analysis

The K562 cells were treated with certain concentrations of Mere15 (0–80 µg/mL) for 24 h. The cells were harvested and ﬁxed in ice-cold 70% (v/v) ethanol for 24 h at 4 °C. The cell pellet was collected by centrifugation at 5000× *g*, resuspended in PBS, and stained with a mixture of RNase (10 μg/mL) and PI (50 μg/mL) in sodium citrate containing 0.5% Triton X-100 for 20 min in the dark. Cell cycle analysis was performed using an Aria FACS flow cytometry system (Bection Dickinson, USA), and cell cycle distribution was analyzed with ModFit LT software (Verity Software House. Inc., USA). 

### 3.8. Immunofluorescence Detection of Cellular Microtubule

Microtubule polymerization in K562 cells was performed as described previously [25]. K562 cells were exposed to certain concentration of Mere15 (0–80 μg/mL) for 24 h. The cells were then harvested and fixed with 4% paraformaldehyde, permeabilized with 0.1% Triton X-100, and blocked with 5% BSA solution. The microtubules were then labeled with mouse monoclonal anti-α-tubulin antibody (1:500, Calbiochem, USA), followed by incubated with the second antibody (FITC-anti-mouse IgG, 1:500, Calbiochem, USA). Cellular microtubules were observed under fluorescence microscopy (Zeiss, Germany).

### 3.9. Tubulin Polymerization Assay

Tubulin (12 μM) was treated with certain concentrations of Mere15 (0–400 μg/mL) in PEM buffer (80 mM PIPES, pH 6.9, 2 mM MgCl_2_, 0.5 mM EGTA, 1 mM GTP, and 5% glycerol (v/v)). Tubulin polymerization was monitored at 37 °C by light scattering at 340 nm using a microplate reader.

### 3.10. Intrinsic Tryptophan Fluorescence of Tubulin

Tubulin (2 μM) was pretreated with certain concentrations of Mere15 (0–300 μg/mL) for 30 min at 37 °C. The intrinsic fluorescence spectra (300–400 nm) were measured using a Cary Eclipse fluorescence spectrophotometer (Varian Inc., USA) with the excitation wavelength was 295 nm.

### 3.11. Hydrophobic Analysis of Tubulin Using Bis-ANS

Tubulin (2 μM) was incubated at 37 °C for 30 min in the absence or presence of certain concentrations of Mere15 (0–300 μg/mL). Bis-ANS (15 μM) was then added, and fluorescence was measured after incubation at 37 °C for 15 min (λ_ex_ = 400 nm, λ_em_ = 430–590 nm) using FlexStation II microplate spectrofluorometer (Molecular Devices, USA).

### 3.12. Data Analysis

One-way ANOVA with Tukey’s post hoc test was used for statistical analysis, and values were expressed as mean ± SD. Differences of *P* < 0.05 were considered statistically significant.

## 4. Conclusions

In conclusion, our present study provides solid evidence that Mere15 could inhibit the growth of K562 cells in a concentration dependent manner, resulted from apoptosis and cell cycle arrest. Mere15 induced overproduction of ROS and disputed the MMP, and therefore resulted in apoptosis in K562 cells. In addition, Mere15 interacted with tubulin, inhibited the polymerization of microtubules and arrested the K562 cells in G_0_/G_1_ phase. In the past decades, thousands of anticancer agents from marine source have been identified. However, most of the agents belong to small chemical molecules. Our study also suggests that the macromolecule polypeptide or proteins, a rich source in marine organisms, could also be important therapeutic candidates for the treatment of cancer diseases. 
